# Sneaker “jack” males outcompete dominant “hooknose” males under sperm competition in Chinook salmon (*Oncorhynchus tshawytscha*)

**DOI:** 10.1002/ece3.869

**Published:** 2013-11-11

**Authors:** Brent Young, David V Conti, Matthew D Dean

**Affiliations:** 1 Molecular and Computational Biology, University of Southern CaliforniaRay R. Irani Building room 304A, 1050 Childs Way, Los Angeles, California, 90089; 2 Department of Preventive Medicine, Keck School of Medicine, University of Southern California2001 N. Soto Street 202S, Los Angeles, California, 90089

**Keywords:** Hooknose, jack, salmon, sexual selection, sneaker male, sperm competition

## Abstract

In a variety of taxa, males deploy alternative reproductive tactics to secure fertilizations. In many species, small “sneaker” males attempt to steal fertilizations while avoiding encounters with larger, more aggressive, dominant males. Sneaker males usually face a number of disadvantages, including reduced access to females and the higher likelihood that upon ejaculation, their sperm face competition from other males. Nevertheless, sneaker males represent an evolutionarily stable strategy under a wide range of conditions. Game theory suggests that sneaker males compensate for these disadvantages by investing disproportionately in spermatogenesis, by producing more sperm per unit body mass (the “fair raffle”) and/or by producing higher quality sperm (the “loaded raffle”). Here, we test these models by competing sperm from sneaker “jack” males against sperm from dominant “hooknose” males in Chinook salmon. Using two complementary approaches, we reject the fair raffle in favor of the loaded raffle and estimate that jack males were ∼1.35 times as likely as hooknose males to fertilize eggs under controlled competitive conditions. Interestingly, the direction and magnitude of this skew in paternity shifted according to individual female egg donors, suggesting cryptic female choice could moderate the outcomes of sperm competition in this externally fertilizing species.

## Introduction

Evolutionary processes have produced a stunning variety of characteristics that appear adaptive for male reproductive success, including morphological weaponry, genitalic, and sperm features, and alternative mating strategies (Andersson 1994). While dominant males fight to secure territory and access to females, many species include “sneaker” males that forego the physiological costs associated with dominance and instead attempt to reproduce surreptitiously. Sneaker males usually encounter numerous obstacles to fertilization, including reduced access to females, and the virtual guarantee that their sperm will be competing with sperm from other males. Nevertheless, sneaking represents an evolutionarily stable strategy under many conditions.

How sneaker males compensate for their apparent reproductive disadvantages is a subject of much interest. Using game theory, Parker (1990b) formalized the “sneak-guard” model to identify conditions where sneaker males represent an evolutionarily stable strategy (Maynard Smith 1982; Gross 1985, 1991, 1996; Parker 1990a,b; Tanaka et al. 2009). Finite resources create a fundamental trade-off between development of precopulatory (i.e., weaponry) versus postcopulatory (i.e., sperm competitive ability) traits (Parker 1990a; Pitcher et al. 2009; Tazzyman et al. 2009; Fitzpatrick et al. 2012). In general, dominant males invest in weaponry that can be used to monopolize access to females, while sneaker males invest in ejaculates to win fertilizations through sperm competition.

Under the sneak-guard model, sneaker males invest in ejaculates via two nonexclusive mechanisms, the “fair raffle” versus the “loaded raffle”. A fair raffle implies that sperm competition outcomes are determined by the relative quantity of competing sperm, and selection favors sneaker males that produce more sperm per unit body mass than dominants. Consistent with this prediction, sneaker males in many different species have larger testes relative to their body mass compared with dominant males (Stockley and Purvis 1993; Gage et al. 1995; Stockley et al. 1997; Taborsky 1998; Simmons et al. 1999; Rasotto and Mazzoldi 2002; Neff et al. 2003; Schulte-Hostedde et al. 2005; Rudolfsen et al. 2006; Montgomerie and Fitzpatrick 2009; Simmons and Fitzpatrick 2012). Under a loaded raffle, selection favors sneaker males that produce higher quality sperm compared with dominant males (Parker 1990a). Sperm quality can include enhanced velocity and/or ATP stores (Taborsky 1998; Uglem et al. 2001; Vladić and Järvi 2001; Burness et al. 2004; Fitzpatrick et al. 2007; Locatello et al. 2007; Pitcher et al. 2009; Vladić et al. 2010; Beausoleil et al. 2012; Tourmente et al. 2013), increased longevity (Smith and Ryan 2010), and/or morphological features (Stockley et al. 1997; Simmons et al. 1999; Balshine et al. 2001; Burness et al. 2004; Snook 2005; Smith and Ryan 2010; Gómez Montoto et al. 2011; Tourmente et al. 2011). Differences in sperm quality can also arise from a male's behavioral adaptations, such as better-timed sperm release close to eggs.

Most direct studies of sperm competition among dominant and sneaker males have been unable to distinguish the fair and loaded raffle models. Fu et al. (2001) estimated that sneaker bluegill males fertilized 78% of embryos when in competition with a dominant male, but it is not clear whether this was due to differences in spawning behavior, ejaculate volume, density, and/or sperm quality. Stoltz and Neff (2006) estimated that sneaker male sperm was nearly twice as competitive as dominant male sperm, but sneaker male sperm were released closer to the female's eggs to mimic natural conditions. Vladić et al. (2010) competed sperm from sneaker and dominant males in Atlantic salmon, finding that sneaker males fertilized 3.6× as many offspring as dominant males after sperm numbers were controlled. Other sperm competition experiments controlled sperm count and distance to female gametes, but competing males were chosen randomly instead of explicitly testing a dominant versus sneaker male (Evans et al. 2003; Gage et al. 2004; Hoysak et al. 2004; Liljedal et al. 2008; Boschetto et al. 2011).

Here, we perform controlled in vitro sperm competition experiments between dominant “hooknose” and sneaker “jack” males in Chinook salmon (*Oncorhynchus tshawytscha*). Using a combination of maximum likelihood, logistic regression, and independent subsampling, we reject the fair raffle in favor of the loaded raffle model, demonstrating that sneaker jack males make competitively superior sperm to dominant males. Although jack males outcompeted hooknoses overall, the magnitude and even the direction of their competitive superiority shifted with individual female egg donor, suggesting females influence the outcomes of sperm competition.

## Materials and Methods

### Study system

Chinook salmon offer an ideal study species for asking whether a sneak-guard system follows the fair or loaded raffle. Young fry leave their natal stream during the smolt and spend the next few years in the open ocean (Healey 1991). As in many salmonids, large dominant “hooknose” males return to their natal streams after 3–7 years, and possess elaborate secondary sexual characteristics such as a kype (the “hooked nose”), a defensive hump, and elongated teeth, which they use to fight for dominance and establish access to nesting females (Gross 1985; Healey 1991; Quinn and Foote 1994; Allen et al. 2007). Sneaker males, referred to as “jacks”, are roughly half the size of hooknose males and do not develop any of these secondary sexual characteristics (Berejikian et al. 2010; Williamson et al. 2010). Instead, jacks take on cryptic coloration and occupy the peripheral edges of rivers, where they wait for hooknose males to begin spawning with females, then dart in and around the spawning pair to release their sperm while avoiding aggressive interactions with dominant males (Heath et al. 1994; Fleming and Reynolds 2004).

Because dominant males vigorously defend nesting females, they are expected to outcompete jack males for access to ova (Rutter 1903; Ginzburg 1972; Gile and Ferguson 1995; Perchec et al. 1998; Hoysak and Liley 2001; Kime et al. 2001; Cosson 2010; Sørum et al. 2011). Consistent with this expectation, sneaker males only sire about 20% of offspring under natural spawning conditions when competing against dominant males (Hutchings and Myers 1988; Jordan and Youngson 1992; Berejikian et al. 2010). However, in spite of their reproductive disadvantages, jacks represent ∼10% of the males in the population, across multiple salmonid species (Myers et al. 1998; Appleby et al. 2003; Carlson et al. 2004; Fleming and Reynolds 2004). In combination with the high heritability of jacking (Heath et al. 2002; Berejikian et al. 2011), these results suggest that sneaking is an evolutionarily stable strategy in this system and that jacks compensate for their disadvantaged mating positions via other mechanisms such as sperm competitive ability.

### Fish selection and gamete collection

Our experimental design represents a trade-off between testing numerous fully independent parents versus multiple observations from the same gamete combinations. We increased the number of observations per sperm-egg combination in order to test for sperm-by-egg interactions. We account for the non-independence of this approach using a variety of statistical methods and subsampling as described below.

A total of five females, five jack males, and five dominant hooknose males (Appendix S1) were collected at the Big Creek Hatchery weir (Oregon Department of Fish and Wildlife) in northwestern Oregon during early October of the 2008 spawning season. Jack males were distinguished from hooknose males based on their smaller size, lack of defensive hump, lack of kype, smaller teeth, and cryptic coloration resembling a female. Only sexually mature fish in good physical condition – without injuries, fungus, and fin wear – were selected.

Prior to gamete collection, fish were wiped dry with paper towels to preclude contamination with water and mucus. Sperm were collected in a beaker by gently bending the male and immediately placed at 4°C. Sperm are quiescent at this stage and do not become active until exposure to water (Kime et al. 2001; Cosson 2010). Females were euthanized and egg masses dissected. Eggs from each female were divided into five approximately equal batches for subsequent exposure to sperm. Sperm count for each male was measured with three independent spermatocrit reads; the ejaculate was centrifuged and the percent of packed sperm taken as a measurement of sperm count per ejaculate (Bouck and Jacobson 1976; Appendix S2). Jack and hooknose sperm are indistinguishable in their sperm head length or width, or flagellum length (Flannery et al. 2013), so spermatocrit measurements are appropriate for comparing sperm counts between males. No formal attempt was made to equalize sperm counts across treatments, but no significant difference was observed between jack and hooknose spermatocrit (*F*_1,20_ = 0.98, *P* = 0.33; Appendix S2). Therefore, paternity skew between male morphs cannot be ascribed to differences in sperm count. In an attempt to minimize experimental noise associated with similar experiments (Gharrett and Shirley 1985; Withler 1988), each jack:hooknose sperm mixture was mixed once, then applied to five different aliquots of female eggs (five total sperm mixtures rather than 25 total sperm mixtures, Table 1).

**Table 1:** Paternity under sperm competition

	Hooknose 1:Jack 1	Hooknose 2:Jack 2	Hooknose 3:Jack 3	Hooknose 4:Jack 4	Hooknose 5:Jack 5	Row sum
Female 1	31:55 (0.36:0.64)	31:49 (0.39:0.61)	39:49 (0.44:0.56)	25:44 (0.36:0.64)	17:29 (0.37:0.63)	143:226 (0.39:0.61)
Female 2	26:35 (0.43:0.57)	18:28 (0.39:0.61)	19:27 (0.41:0.59)	32:45 (0.42:0.58)	10:36 (0.22:0.78)	105:171 (0.38:0.62)
Female 3	47:44 (0.52:0.48)	37:47 (0.44:0.56)	14:28 (0.33:0.67)	27:41 (0.40:0.60)	39:29 (0.57:0.43)	164:189 (0.46:0.54)
Female 4	42:35 (0.55:0.45)	38:8 (0.83:0.17)	32:14 (0.70:0.30)	7:39 (0.15:0.85)	23:45 (0.34:0.66)	142:141 (0.50:0.50)
Female 5	28:17 (0.62:0.38)	22:47 (0.32:0.68)	31:14 (0.69:0.31)	10:59 (0.14:0.86)	39:50 (0.44:0.56)	130:187 (0.41:0.59)
Column sum	174:186 (0.48:0.52)	146:179 (0.45:0.55)	135:132 (0.51:0.49)	101:228 (0.31:0.69)	128:189 (0.40:0.60)	684:914 (0.43:0.57)

Number of embryos sired by hooknose:jack (proportions in parentheses).

### Experimental crosses/mating scheme

To include male–female interaction terms, a variant of the North Carolina II breeding design (Comstock and Robinson 1948) was employed, with each of five rows representing eggs from one female, and each of five columns representing a unique mixture of sperm from one hooknose and one jack male (5 mL sperm from one hooknose male, 5 mL from one jack male, 10 males total; Table 1). Sperm combinations were mixed by gently swirling a beaker for 5 min. Approximately 500 eggs from each female were placed on one side of a new beaker and 1 mL of the sperm mixture on the opposite side. Gametes were mixed with the turbulent addition of 1000 mL of natural temperature Big Creek river water and swirled for 10 sec. The egg–sperm mixtures were allowed to stand for 5 min before transfer to Heath tray incubators at the Big Creek Hatchery facilities. Fertilized eggs were reared according to standard hatchery practices, with each individual replicate in a separate tray. Mortalities were removed and collected each week until the eyed stage (approximately 40 days postfertilization), at which time, all eggs were euthanized and preserved for subsequent genetic analysis. Mortality was so low (<5%) that even if one male type sired all the dead eggs in a tray, our conclusions below would not change.

### Genetic analysis/parentage assignment

DNA was extracted from muscle tissue taken from the 15 possible parents and from the heads of individual embryos using an Epicentre MPC extraction kit, following the manufacturer's instructions. Three microsatellite loci – OTS213 (Greig et al. 2003), OTS107 (Nelson and Beacham 1999), and RT212 (Spies et al. 2005) – allowed unambiguous paternity assignment in any given cross (Appendix S1). One primer in each pair was dyed with HEX or FAM for downstream scoring. PCR amplifications consisted of 2 min of denaturation at 94°C, followed by 35 cycles of 30 sec denaturation (94°C), 30 sec annealing (each locus-specific temperature), 40 sec elongation (72°C), and a final 5 min extension at 72°C. Genotyping was performed by the University of Arizona Genetics Core on an ABI Prism 3730 DNA Analyzer (Applied Biosystems, Grand Island, NY). A total of 1598 embryos were genotyped, with an average 63.9 embryos genotyped from each of the 25 combinations of sperm and eggs (range = 42–91, standard deviation = 17.1, Table 1).

### Statistical analyses

We employed two distinct methods to test for competitive differences between jack male sperm and hooknose male sperm. The first was a maximum-likelihood method that considers each brood as an independent observation, and the second was a logistic regression that considers each embryo as an independent observation. For the maximum-likelihood approach, we also subsampled totally independent datasets from the full dataset. There are 120 different ways to sample the 5 × 5 experimental design where no rows or columns are shared.

#### Maximum likelihood

Neff and Wahl (2004) developed a maximum-likelihood method to test whether sperm competition outcomes follow fair or loaded raffles. For each of 25 broods (Table 1), paternity outcomes follow:


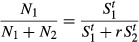


where *N*_1_ and *N*_2_ are the numbers of offspring sired by male 1 and male 2 in a brood, respectively; *S*_1_ and *S*_2_ are the numbers of sperm transferred by male 1 and male 2 (taken as the average of the three spermatocrit values taken per male, Appendix S2), respectively; *r* is the competitive ability of the second male's relative to the first male's sperm; *t* is a measure of the economy of scale to sperm number. Essentially, *t* measures whether the returns on transferring additional sperm follow a linear trend. If *t* = 0, then the above equation reduces to 1/(1 + *r*), indicating that sperm competition outcomes are independent of relative sperm number and determined only by *r*. An individual that makes higher quality sperm gains less per additional sperm transferred if 0 < *t* < 1, but gains disproportionately more if *t* > 1. The method optimizes *r* and *t* across the entire set of broods and estimates 95% confidence intervals through permutation (Neff and Wahl 2004). These confidence intervals were used to test the fair raffle model, where *r* = 1 (no differences in sperm competitive ability) and *t* = 1 (sperm competition outcomes related only to *S*_1_ relative to *S*_2_ and *r*), as well as the sperm-independent model, where *t* = 0. Because spermatocrit numbers did not significantly differ between jack and hooknose males (Appendix S2), our study was probably underpowered to uncover differences due sperm quantity. However, our primary goal was to test the null hypothesis *r* = 1, the prediction under a fair raffle. We applied the maximum-likelihood method to the entire dataset, as well as each of the 120 independent subsamples.

#### Logistic regression

A second method used logistic regression to model the log odds of the probability that a jack male sired an embryo:


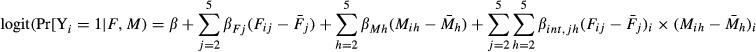


*Y*_*i*_ is a variable indicating if offspring *i* was sired by a jack (*Y*_*i*_ = 1) or hooknose male (*Y*_*i*_ = 0), and *F*_*ij*_ and *M*_*ih*_ are indicator variables denoting the contributing female *j* or male sperm mixture *h*, respectively. It should be emphasized that *M* refers to a single sperm mixture from two males. These variables were mean-centered to allow the *expit(α)* to equal the overall probability of a jack in the sample. Each *β* represented the log odds ratio and a Wald test used to determine whether a factor significantly affected this ratio.

We tested the fit of the data to different models to understand the effects of male and female variables on the probability an offspring was sired by a jack male. Model 1 was a null model that simply calculated the overall mean *Y*_*i*_, without any variables. Model 2, Model 3, and Model 4 added *M*_*ih*_, *F*_*ij*_, or both, respectively, to test whether the identity of the female egg donor and/or male sperm mixture influenced *Y*_*i*_. Model 5 added an interaction between the sexes. Models were compared using a likelihood ratio test (LRT). All tests were performed with customized Python (http://www.python.org) and R (http://www.r-project.com) scripts.

### Skewed paternity, sex ratio, and growth rates

Strong paternity skew could be correlated with sex ratio if sex-linked meiotic drive reduced the ability of one male to compete. We tested for sex skew by amplifying X- and Y-specific regions (Devlin et al. 1994) from a subset of embryos from two gamete combinations that revealed highly skewed paternity (Hooknose 2:Jack 2+ Female 4 and Hooknose 4:Jack 4+ Female 4, Table 1).

Strong paternity skew could also be correlated with differences in embryonic developmental rate if cryptic female choice yielded offspring genotypes that grew fast. In salmonids, there are paternal and maternal contributions to egg size and egg metabolic rate (Pakkasmaa et al. 2001, 2006). Although not a primary objective, we tested for differential growth rate, we weighed embryo + yolk from a subset of embryos from four gamete combinations with skewed paternity (Hooknose 2:Jack 2+ Female 4, Hooknose 2:Jack 2+ Female 5, Hooknose 4:Jack 4+ Female 3, and Hooknose 4:Jack 4+ Female 5). All tests were performed with customized Python (http://www.python.org) and R (http://www.r-project.com) scripts.

## Results

### Jack males outcompeted hooknose males

Because we genotyped loci known to discriminate competing males (Appendix S1), all 1598 embryos that were genotyped were scored unambiguously for paternity.

#### Maximum likelihood

The methods of Neff and Wahl (2004) rejected the fair raffle model (*r* = 1 and *t* = 1). Specifically, jack sperm were estimated to be *r* = 1.34× as competitive as hooknose sperm, significantly different than *r* = 1 (*P* < 0.0001) and very consistent with the 1.36× estimated from logistic regression analyses presented below. *t* was estimated to be <10^−12^, which was not significantly different from either *t* = 0 or *t* = 1 (*P* = 0.99, *P* = 0.50, respectively).

From the 5 × 5 Table 1, there are 120 possible ways to sample five cells with no rows or columns in common. Of these, 82 rejected the null hypothesis *r* = 1 (*P* < 0.05), in favor of the alternative that jack males were superior under controlled sperm competition. The average ± standard deviation *r* in these cases was 1.52 ± 0.25. In contrast, only one independent subsample favored the alternative that hooknose males were competitively superior.

#### Logistic regression

Overall, an embryo had a probability of 0.576 of being sired by a jack male, significantly different from the null expectation of 0.50 (*P* = 3.97 × 10^−8^, Table 2). In other words, jack sperm were 0.576/(1 − 0.576) = 1.36× as competitive as hooknose sperm, a number that is very similar to the maximum-likelihood estimates presented above. Female 4 deviated significantly from background, with a preference for hooknose sperm (*P* = 0.007, Table 2). Two sperm mixtures were significantly more jack-skewed than background. Jack 4 sired 0.711 of the embryos when in competition with Hooknose 4, and Jack 5 sired 0.601 of the offspring when in competition with Hooknose 5; both were significantly higher than background (*P* = 2.05 × 10^−7^, *P* = 0.014, respectively, Table 2).

**Table 2:** Coefficients estimated from full model (Model 5)

Coefficients (Model parameter)	Estimate	SE	*P* (sired by Jack)	*z*-value	Pr (>|*z*|)	Significance (*P*)
Intercept	0.306	0.056	0.576	5.492	3.97E−08	≤0.001
Female 2 (F_2_)	0.057	0.171	0.514	0.333	0.739	
Female 3 (F_3_)	−0.306	0.157	0.424	−1.953	0.051	
Female 4 (F_4_)	−0.492	0.184	0.379	−2.674	0.007	≤0.01
Female 5 (F_5_)	−0.159	0.172	0.460	−0.924	0.355	
Hooknose 2:Jack 2 (M_2_)	0.072	0.166	0.518	0.432	0.666	
Hooknose 3:Jack 3 (M_3_)	−0.075	0.173	0.481	−0.437	0.662	
Hooknose 4:Jack 4 (M_4_)	0.900	0.173	0.711	5.195	2.05E−07	≤0.001
Hooknose 5:Jack 5 (M_5_)	0.409	0.167	0.601	2.456	0.014	≤0.05
Female 2 * Hooknose 2:Jack 2 (F_2_ * M_2_)	0.260	0.511	0.565	0.509	0.611	
Female 3 * Hooknose 2:Jack 2 (F_3_ * M_2_)	0.421	0.442	0.604	0.952	0.341	
Female 4 * Hooknose 2:Jack 2 (F_4_ * M_2_)	−1.260	0.554	0.221	−2.275	0.023	≤0.05
Female 5 * Hooknose 2:Jack 2 (F_5_ * M_2_)	1.374	0.514	0.798	2.671	0.008	≤0.01
Female 2 * Hooknose 3:Jack 3 (F_2_ * M_3_)	0.399	0.503	0.599	0.793	0.428	
Female 3 * Hooknose 3:Jack 3 (F_3_ * M_3_)	1.104	0.498	0.751	2.219	0.026	≤0.05
Female 4 * Hooknose 3:Jack 3 (F_4_ * M_3_)	−0.299	0.502	0.426	−0.597	0.551	
Female 5 * Hooknose 3:Jack 3 (F_5_ * M_3_)	0.049	0.543	0.512	0.091	0.928	
Female 2 * Hooknose 4:Jack 4 (F_2_ * M_4_)	0.052	0.483	0.513	0.107	0.915	
Female 3 * Hooknose 4:Jack 4 (F_3_ * M_4_)	0.492	0.468	0.621	1.052	0.293	
Female 4 * Hooknose 4:Jack 4 (F_4_ * M_4_)	1.908	0.578	0.871	3.301	0.001	≤0.001
Female 5 * Hooknose 4:Jack 4 (F_5_ * M_4_)	2.282	0.570	0.907	4.005	0.000	≤0.001
Female 2 * Hooknose 5:Jack 5 (F_2_ * M_5_)	1.023	0.582	0.736	1.758	0.079	
Female 3 * Hooknose 5:Jack 5 (F_3_ * M_5_)	−0.191	0.498	0.452	−0.384	0.701	
Female 4 * Hooknose 5:Jack 5 (F_4_ * M_5_)	0.893	0.512	0.709	1.745	0.081	
Female 5 * Hooknose 5:Jack 5 (F_5_ * M_5_)	0.787	0.533	0.687	1.476	0.140	

Significance indicates factors that differed from an overall null model.

A model including sperm aliquot as a fixed effect explained the data significantly better than a model ignoring it (Model 2 vs. Model 1, χ^2^ = 32.70, df = 4, *P* = 10^−6^, Table 3), as did a model including female donor (Model 3 vs. Model 1, χ^2^ = 13.63, df = 4, *P* = 0.01), showing that the general superiority of jack male sperm was not uniform across sperm aliquot or egg donor. A model including both male and female fit the data significantly better than models with only male (Model 4 vs. Model 2, χ^2^ = 13.29, df = 4, *P* = 0.01) or only female (Model 4 vs. Model 3, χ^2^ = 32.37, df = 4, *P* = 10^−6^, Table 3). Taken together, these results suggest that both sperm mixture and egg donor influence the outcomes of sperm competition.

**Table 3:** Comparison of logistic regression models using likelihood ratio test

Model number	Variables added	Model architecture	Residual deviance	df	Model comparisons (LRT)
1	Null	*Y* ∼ 1	2182.1	1597	
2	Male	*Y* ∼ Male	2149.4	1593	2 vs. 1: χ^2^ = 32.70, df = 4, *P* = 10^−6^
3	Female	*Y* ∼ Female	2168.4	1593	3 vs. 1: χ^2^ = 13.63, df = 4, *P* = 0.01
4	Both	*Y* ∼ Male + Female	2136.1	1589	4 vs. 2: χ^2^ = 13.29, df = 4, *P* = 0.01
					4 vs. 3: χ^2^ = 32.37, df = 4, *P* = 10^−6^
5	Interaction	*Y* ∼ Male + Female + interaction	2042.3	1573	5 vs. 4: χ^2^ = 93.82, df = 16, *P* = 10^−13^

Significant LRT signifies a better fit to the data in the more complex model.

LRT, likelihood ratio test.

### Females may influence the outcomes of sperm competition

In the logistic regression framework, a model including an interaction term between sperm mixture and egg donor fit the data significantly better than a model with only additive male and female effects (Model 5 vs. Model 4, χ^2^ = 93.82, df = 16, *P* = 10^−13^, Table 3). This effect is best illustrated by the Hooknose 2:Jack 2 sperm mixture. Jack 2 sired 0.798/(1 − 0.798) = 3.95× more offspring than Hooknose 2 when combined with Female 5 (*P* = 0.008, Table 2) but 0.221/(1 − 0.221) = 0.28× as many offspring as Hooknose 2 when combined with Female 4 (*P* = 0.023, Table 2). Thus, the outcomes of sperm competition between two particular males depended upon female genotype.

An alternative explanation to explain the sperm-by-egg interaction term is that random effects were very high. However, we emphasize that the same exact sperm aliquot was delivered across the eggs from five females. Therefore, random effects are unlikely to explain the sperm-by-egg interaction term.

### Paternity skew was not correlated with sex ratio or growth rates

There was no evidence that paternity skew was related to meiotic drive of the sex chromosomes. For the Hooknose 2:Jack 2+ Female 4 combination, 11 males and nine females were sired by the hooknose male while two males and one female were sired by the jack male. For the Hooknose 4:Jack 4+ Female 4 combination, three males and two females were sired by the hooknose male while eight males and nine females were sired by the jack male. Pooling these data revealed 19 male and 18 female offspring sired by the winning male, compared with five males and three females sired by the losing male (Fisher's Exact Test, *P* = 0.71).

There was no evidence that growth rate of embryos correlated with winning sires. Pooling across the four gamete combinations surveyed in this manner, 69 embryos sired by the winning male (median embryo:total egg weight = 0.188 g) were not significantly different from the 23 embryos sired by losing males (median embryo:total egg weight = 0.187 g, Mann–Whitney *P* = 0.66).

## Discussion

Sneak-guard mating systems are prevalent among animal species, but the mechanisms by which sneaker males maintain reproductive fitness remain incompletely characterized (Gross 1996; Taborsky 1998). Here, we reject the fair raffle model, showing that sperm from sneaker jack males were competitively superior to sperm from dominant hooknose males in controlled in vitro fertilization experiments. Thus, sperm competition outcomes in Chinook salmon are best explained as a loaded raffle (Parker 1990a), helping to explain the stability of sneaker males in this system.

Several hypotheses could explain the general superiority of jack sperm over hooknose sperm. First, jack sperm swim faster than hooknose sperm (Flannery et al. 2013), and sperm velocity is a primary determinant of fertilization success in sperm competition in numerous fish species (Burness et al. 2004; Gage et al. 2004; Liljedal et al. 2008; Rudolfsen et al. 2008; Boschetto et al. 2011; Evans et al. 2013) and other external fertilizers (Levitan 1993, 1996, 2000; Kupriyanova and Havenhand 2002; Marshall et al. 2002). The speed with which sperm can locate an egg is important. In Sockeye salmon, over 80% of eggs are fertilized within 5 sec of gamete activation (Hoysak and Liley 2001) and sperm generally live <1 min upon activation (Kime et al. 2001; Cosson 2010).

Second, the exact combination of sperm and egg proteins can influence fertilization in external fertilizers (Vacquier 1998; Swanson and Vacquier 2002; Bernasconi et al. 2004). In salmon, sperm bind to “sperm guidance” glycoproteins as they traverse through the mucus layer and into the micropyle, which is the site of fertilization (Yanagimachi et al. 1992; Iwamatsu et al. 1997; Mengerink and Vacquier 2001), and it is possible that jack and hooknose sperm respond differently to egg proteins. Different combinations of male and female proteins translate into differential fertilization rates in many externally species (Gaffney et al. 1993; Palumbi 1999; Boudry et al. 2002; Evans and Marshall 2005; Geyer and Palumbi 2005; Marshall and Evans 2005; Levitan and Ferrell 2006; Levitan and Stapper 2010; Levitan 2012).

Third, if inbreeding avoidance mechanisms exist in Chinook salmon, they are likely to favor jack male sperm. Spawning hooknose males and females could have been born in the same river and same year, and could be close relatives. Because jack males return to spawn at least 1 year earlier than females of their same cohort, they should be less genetically related to currently spawning females than dominant hooknose males. In guppies, a male's sperm displayed higher velocity in the presence of ovarian fluid from an unrelated female, suggesting a mechanism by which females may bias paternity toward unrelated males (Gasparini and Pilastro 2011; Gasparini et al. 2012). Salmonid sperm motility is influenced by female ovarian fluid (Rosengrave et al. 2008; Flannery 2011; Yeates et al. in press). Generally, however, dominant male sperm swim faster in female ovarian fluid compared with jack males (the opposite trend is observed in river water; Flannery 2011). Mechanisms of inbreeding avoidance, if they exist, may be more complicated than simple predictions based on interactions between sperm and ovarian fluid, however. For example, genetic variation at the major histocompatibility locus has been shown to affect gamete interactions (Skarstein et al. 2005; Yeates et al. 2009).

Our finding that jack males make competitively superior sperm calls into question a common viewpoint that jack males are less fit than dominant males and are “making the best of a bad situation”. Reichard et al. (2007) reviewed theoretical and empirical examples where females might actually benefit from allowing sneaker males to fertilize their eggs, including increased genetic diversity in their offspring. Interestingly, female bluegill spawn more eggs when sneaker males are present, and sneaker males in that system also fertilize a disproportionate share of eggs (Fu et al. 2001). This could be an example whereby female choice favors fertilization by sneaker males. In fact, precocious sexual maturity might be a general indication that sneaker males are more genetically robust to environmental stresses, a very different viewpoint than one that assumes they are poor quality individuals. Interestingly, over-feeding in hatcheries often leads to increased rates of jacking, consistent with this interpretation.

We set out to elucidate the apparent stability of jack males in the mating ecology of Chinook salmon. Using controlled in vitro sperm competition experiments, we demonstrated that sneaker jack males outcompete dominant hooknose males via a loaded raffle. Therefore, jacks appear to invest disproportionately in sperm quality. Two distinct methods estimated that jack sperm were ∼1.3× as competitive as hooknose sperm. In addition, female egg donors affected sperm competition outcomes, though the underlying mechanisms remain unknown. Future investigations into the molecular basis of the loaded raffle will lead to greater insight into the stability of this sneaker male morphotype in Chinook salmon.

## Supporting Information

**Appendix S1.** Parental genotypes and phenotypes.

**Appendix S2.** Spermatocrit data from all participating males.
